# Emergence of a Novel Canine Distemper Virus Variant in Urbanized Free-Ranging Marmosets (*Callithrix penicillata*)

**DOI:** 10.1155/tbed/4818076

**Published:** 2025-08-15

**Authors:** Tais Meziara Wilson, Davi Emanuel Ribeiro de Sousa, Isabel L. Macêdo, Mizael Machado, Gabriela Rodrigues de Toledo Costa, Pamela Fair, Jana M. Ritter, Hannah A. Bullock, Marlene DeLeon-Carnes, Elizabeth Lee, Pedro Henrique de Oliveira Passos, Daniel Garkauskas Ramos, Alessandro Pecego Martins Romano, Sandro Patroca da Silva, Livia Medeiros Nevs Casseb, Livia Caricio Martins, Luis Janssen Maia, Bergmann Morais Ribeiro, Eduardo Mauricio Mendes de Lima, Cristiano Barros de Melo, Márcio Botelho de Castro

**Affiliations:** ^1^Veterinary Pathology and Forensic Laboratory, University of Brasília, Brasília, Brazil; ^2^Plataforma de Salud Animal, Instituto Nacional de Investigacion Agropecuaria (INIA), Tacuarembó, Uruguay; ^3^Environmental Health Surveillance Directorate of the Federal District, Brazilian Ministry of Health, Brasilia, Brazil; ^4^Infectious Diseases Pathology Branch, Centers for Disease Control and Prevention, Atlanta, Georgia, USA; ^5^Technical Group on Arbovirus Surveillance, Brazilian Ministry of Health, Brasilia, Brazil; ^6^Section of Arbovirology and Hemorrhagic Fevers, Instituto Evandro Chagas, Pará, Brazil; ^7^Laboratory of Baculovirus, Cell Biology Department, University of Brasília, Brasília, Brazil; ^8^Graduate Program in Animal Sciences, University of Brasília, Brasília, Brazil

**Keywords:** canine distemper virus, marmoset, metagenomic, One Health, pathology, phylogeny

## Abstract

The black-tufted marmoset (*Callithrix penicillata*), commonly found in urban areas of Central Brazil, is vulnerable to pathogen spillover from domestic animals and humans. Here, we report an outbreak of natural canine distemper virus (CDV) infection among urbanized free-ranging black-tufted marmosets. Five fatalities occurred in marmosets living in a neighborhood with unvaccinated dogs. Clinically, affected marmosets had lethargy, ataxia, mucocutaneous ulcerations, and crusting lesions. Postmortem findings included epithelial erosions, interstitial pneumonia, bronchopneumonia, and suppurative myocarditis, frequently associated with secondary bacterial infections. Immunohistochemistry (IHC) confirmed the presence of CDV antigen in multiple organs, and secondary bacterial infections were common, involving species, such as *Bordetella*, *Haemophilus*, and *Streptococcus*. Transmission electron microscopy (TEM) demonstrated paramyxovirus-like inclusion bodies and metagenomic sequencing identified a novel CDV variant. Phylogenetic analyses placed this strain within the Europe 1/South America 1 lineage, closely related to domestic dog-derived strains from the region. Comparative H gene analysis uncovered unique R519I substitutions in the CDV marmoset variant, suggesting potential for cross-species adaptation. This study provides evidence that CDV can naturally infect free-living New World primates, with possible implications for animal health, conservation, and interspecies transmission. These findings highlight the vulnerability of urban wildlife to CDV spillover from domestic dogs and emphasize the importance of monitoring pathogen transmission at the human–animal interface from a One Health perspective.

## 1. Introduction

The black-tufted marmoset is widely distributed across Brazil, including human-inhabited areas, and is susceptible to several human pathogens, such as measles virus, herpes simplex viruses, parainfluenza virus 1, and yellow fever (YF) virus [1]. These examples underscore the potential for pathogen exchange between humans and marmosets, reinforcing the need for infectious disease surveillance in this species under the One Health concept [2].

The *Paramyxoviridae* family, composed of enveloped, negative-sense, single-stranded RNA viruses, includes the *Morbillivirus* and *Henipavirus* genera, both of which have caused major human outbreaks, including measles, Hendra, and Nipah viruses. These viruses are notable for their severe pathogenicity and broad host and geographic ranges [3]. Canine distemper virus (CDV), a *Morbillivirus*, is closely related to measles virus [4]. Its approximately 15,000 nucleotides genome encodes six proteins, among which the hemagglutinin (H) protein is the most variable and crucial for host cell binding [5]. In dogs, CDV primarily spreads via the respiratory route, initially targeting immune cells by binding to the SLAM receptor, and subsequently infecting epithelial cells through the Nectin-4 receptor, leading to systemic viremia [6].

CDV infects a wide range of species and interspecies transmission has been increasingly reported, likely due to variability in the H protein [7, 8]. Infections in rhesus macaques and neotropical primates of the genus *Callithrix* [9, 10] raise concerns about their zoonotic potential. The COVID-19 pandemic heightened awareness of viruses crossing species barriers. Factors, such as CDV occurrence in primates, respiratory transmission, presymptomatic shedding in ferrets, and declining measles vaccination rates [11] underscore the need for further research.

This study reports the epidemiological, histopathological, immunohistochemical, and phylogenetic features of a novel CDV variant infection in black-tufted marmosets from a peri-urban area in Brasília, Brazil.

## 2. Materials and Methods

### 2.1. Case Samples and Field Investigation

Between June 8 and 28, 2018, five unexplained deaths of free-living black-tufted marmosets (*Callithrix penicillata*) occurred in Brasília, Brazil. Postmortem examinations were conducted at the Regional Reference Laboratory of the Brazilian Ministry of Health for YF Diagnosis as part of the National YF Surveillance Program. Frozen liver and kidney samples were sent to the Instituto Evandro Chagas (IEC) in Belém, Pará, for RNA extraction and YF RT-PCR [12]. Immunohistochemistry (IHC) for YF was performed at the Veterinary Pathology Laboratory of the University of Brasília. All samples tested negative for YF by both PCR and IHC.

Marmosets NHP1, NHP4, and NHP5 were found dead after being observed ill by local residents. NHP2 and NHP3 were submitted to the University of Brasília's Wildlife Medicine Sector for clinical care, where they later died.

### 2.2. Necropsy and Histopathology

All five marmosets underwent necropsy. Tissue samples (brain, spinal cord, lungs and trachea, heart, spleen, lymph nodes, liver, gallbladder pancreas, tongue, stomach, intestines, thyroids, adrenal glands, kidneys, urinary bladder, genital tract, facial haired skin, oral, and ocular mucocutaneous junctions) were fixed in 10% neutral-buffered formalin, processed routinely, embedded in paraffin, sectioned, and stained with hematoxylin and eosin (H&E) for histopathologic evaluation. Samples with histological evidence of bacterial infection (e.g., suppurative inflammation or visible bacteria) were stained using the Lillie–Twort Gram method.

### 2.3. IHC

Formalin-fixed paraffin-embedded (FFPE) tissues (central nervous system (CNS), lungs, spleen, lymph nodes, liver, tongue, stomach, adrenal glands, kidneys, urinary bladder, and skin) were submitted to the CDC's Infectious Diseases Pathology Branch (IDPB) in Atlanta, GA, for CDV detection via polymer-based colorimetric IHC using a polyclonal anti-CDV antibody (VMRD, 1:500 dilution). Detection was performed using a Mach 4 Universal AP Polymer Kit (Biocare Medical) and Permanent Red Chromogen (Cell Marque/Millipore Sigma).

For bacteria-positive cases by L-T Gram staining, IHC was conducted using specific antibodies: anti-*Streptococcus pneumoniae* (Thermo Fisher, cross-reacts with other *Streptococcus* spp.), anti-*Klebsiella pneumoniae* (Thermo Fisher, cross-reacts with *E. coli*, *Haemophilus influenzae*, and *Pseudomonas* spp.), anti-*Bordetella pertussis* (CDC, also detects *B. bronchiseptica*) [13], anti-*H. influenzae* (Fisher Scientific), and anti-*Staphylococcus aureus* (Meridian Life Science).

### 2.4. PCR Assays for Bacterial Agents

For marmosets with bacteria identified by IHC, DNA was extracted from 16 μm FFPE lung sections at IDPB. DNA quality was verified by amplifying the nuclear histone H4 gene (211 bp) and mitochondrial 12S rRNA gene (456 bp). Samples were then subjected to PCR targeting the 16S rRNA gene, specifically for Gram-positive and Gram-negative bacteria [14]. Positive amplicons were sequenced via Sanger sequencing and analyzed using NCBI's BLAST tool.

### 2.5. Electron Microscopy Examination

Tissues with abundant CDV immunostaining were processed for transmission electron microscopy (TEM) at IDPB [15]. Samples were postfixed in 1% osmium tetroxide, en bloc stained with 4% uranyl acetate, dehydrated in graded ethanol and acetone, embedded in Epon-Araldite resin, sectioned (~50 nm), stained with uranyl acetate and lead citrate, and examined with a Tecnai Biotwin electron microscope.

### 2.6. Viral Genome Sequencing and Evolutionary Analyses

Metagenomic sequencing was conducted at IEC using RNA extracted from pooled liver and kidney tissues of each marmoset. At the time, the cause of death was unknown, so we chose an unbiased metagenomic approach to comprehensively detect any potential pathogens present and identify the possible cause of mortality. Comparative samples were obtained from a dog (*Canis familiaris* CA 583) and a crab-eating fox (*Cerdocyon thous* CA 584) that had been previously diagnosed with CDV and necropsied at the University of Brasília.

cDNA synthesis was performed using SuperScript VILO MasterMix and NEBNext Second Strand Synthesis kits, followed by purification with PureLink PCR Purification Kit. Libraries were prepared using the SureSelectQXT Whole Genome Library Prep kit and sequenced on the Illumina NextSeq platform at IEC. Reads were assembled using IDBA-UD v1.1.1 [16] and SPAdes v3.12.0 [17], and viral contigs were identified by aligning them against the RefSeq viral protein database using DIAMOND [18]. Genome annotation was performed using Geneious 7.1.8 [19].

To investigate potential substitutions in the H protein sequences of CDV variants, H, V, F, and P protein sequences from marmoset CDV genomes were aligned with reference sequences from *Macaca mulatta* (HM852904.1), *M. fascicularis* (AB687721.2, AB687720.2), and dog (AB753775.1) using MAFFT v7.489 [20]. Alignments were visualized using Texshade [21]. Genome sequences were deposited in GenBank (Accession Numbers: PP847352, PP847351, PP847348, PP847350, and PP847349).

### 2.7. CDV Phylogeny

Two datasets were used to infer CDV phylogeny based on the H gene. For the broad dataset, 665 nonredundant CDV H gene sequences (1500–1900 nucleotides) were retrieved from GenBank (excluding laboratory variants) using the query: (“Canine morbillivirus”[Organism] OR CDV [All Fields]; AND H [All Fields]) (NOT “laboratory”[All Fields] AND “1500”[SLEN]: “1900”[SLEN]) (Accessed on 08/24/2023). We have also added a H sequence from a dog-derived virus obtained in 2019, thus being contemporary to the ones in our study (Federal District, (Accession Number: MW460905) [22]. Sequences were aligned using MAFFT v7.489, and a maximum likelihood (ML) tree was generated with IQ-TREE2 v2.2.5, employing 10,000 ultrafast bootstraps and the TVM + F+I+R4 model selected by ModelFinder [23]. The tree was midpoint rooted and visualized using iTOL (https://itol.embl.de/) [24] and edited in GIMP (https://www.gimp.org/). Host silhouettes were sourced from PhyloPic (www.phylopic.com).

To investigate amino acid differences, we aligned H, V, F, and P protein sequences from the marmoset- and fox-derived CDV strains with reference sequences from old-world primates (Genbank: HM852904.1, AB687721.2, and AB687720.2), the virus from the coinfected marmoset in the northwest [10], a 2019 dog virus from the Federal District (MW460905) [22] and the highly similar viral H gene from a infected dog in Mato Grosso State (MT119973). For finer strain classification, a second ML tree was constructed using H gene sequences [24] with the GTR + F+I+R2 model. A pairwise distance matrix was also generated to compare CDV lineages.

## 3. Results

### 3.1. Case Histories, Clinical Signs, and Epidemiological Investigation

Five black-tufted marmoset (*Callithrix penicillata*) fatalities occurred during an outbreak in a residential urban area of Brasília, Brazil (15°51′38.9"S 47°58′39.4"W), characterized by homes with large tree-filled yards and the presence of domestic and stray dogs, none of which were vaccinated against CDV. According to local residents, a group of approximately 12 marmosets regularly foraged on the ground, occasionally entering homes. Residents reported observing the deaths of NHP 1, 4, and 5, as well as interactions between free-ranging marmosets and unvaccinated domestic dogs, including instances where the marmosets were seen feeding on dog food.

NHP 1 and 4 appeared lethargic and ataxic shortly before being found dead. NHP 5 developed lethargy and multifocal facial skin erosions/ulcerations, pustules, and scabs and died within 3 days. NHP 2, referred for clinical care, presented with facial, labial, and lingual ulcers and erosions, along with stupor, blindness, and seizures. Despite intensive care, it died within 1 day. NHP 3 had similar lesions and lethargy and died during evaluation. No other marmosets from the group were seen in the area after the outbreak (Table 1).

### 3.2. Pathological Findings

**Figure 1:**
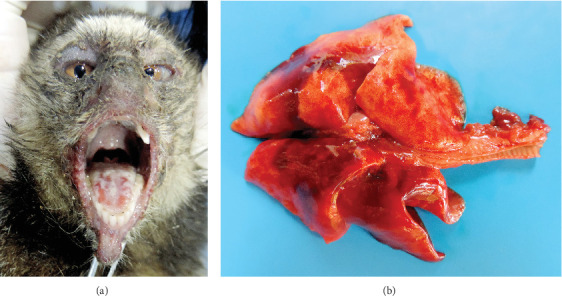
Gross findings of fatal CDV infection in free-ranging urbanized black-tufted marmosets. (A) Tongue, NHP 3. Multifocal ulcers and white plaques on the dorsal surface. (B) Lungs, NHP 4. Heavy, firm, and wet aspect with multifocal mottled dark red areas.

Gross lesions were similar in all five marmosets and included erythematous maculopapular rashes on the nose, lips, and periocular region; multifocal to coalescent white plaques, erosions, and small ulcers on the tongue (Figure 1A); and heavy, firm, and wet lungs with cranioventral consolidation (Figure 1B). Submandibular lymph nodes and spleens were enlarged (Table 1).

**Figure 2:**
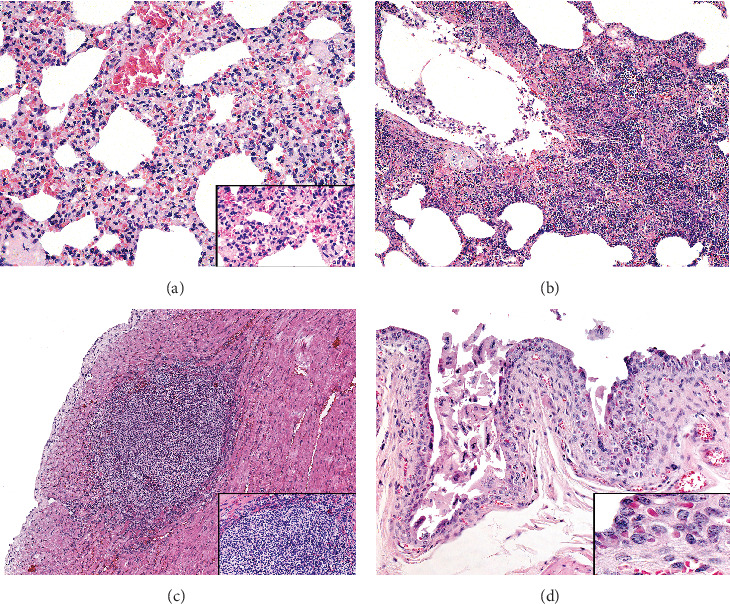
Histopathological findings of fatal CDV infection in free-ranging urbanized black-tufted marmosets. (A) Lung, NHP 1. Interstitial pneumonia with a marked thickness of alveolar septa (H&E, objective, 20 ×). Inset. High-magnification image of mononuclear interstitial inflammatory infiltration. (B) Lung, NHP 5. Bacterial suppurative bronchopneumonia (H&E, objective, 10 ×). (C) Heart, NHP 3. Microabscess within the myocardium (objective, 10 ×). Inset. High-magnification image of suppurative inflammatory infiltrate. (D) Urinary bladder, NHP 2. Numerous intracytoplasmic viral inclusions in the urinary epithelium (H&E, objective, 20 ×). Inset. High-magnification image showing viral inclusions within the urothelium.

Histologically, the skin had hyperkeratotic and acanthotic neutrophilic dermatitis with multifocal ulceration and hemorrhage. The tongue and labial mucosa had parakeratotic hyperkeratosis and necroulcerative glossitis accompanied by moderate submucosal lymphoplasmacytic inflammation. Pulmonary lesions ranged from mild to moderate mononuclear interstitial inflammation (Figure 2A) with syncytial cells to severe necrotizing fibrinosuppurative bronchopneumonia (Figure 2B), characterized by edema and neutrophilic infiltrates containing bacterial colonies. Suppurative myocarditis (Figure 2C), with cardiomyocyte necrosis and intralesional bacteria, was observed in two marmosets, suggesting sepsis. No histological findings were observed in the CNS, including the brain and spinal cord, or in any other organs evaluated.

**Figure 3:**
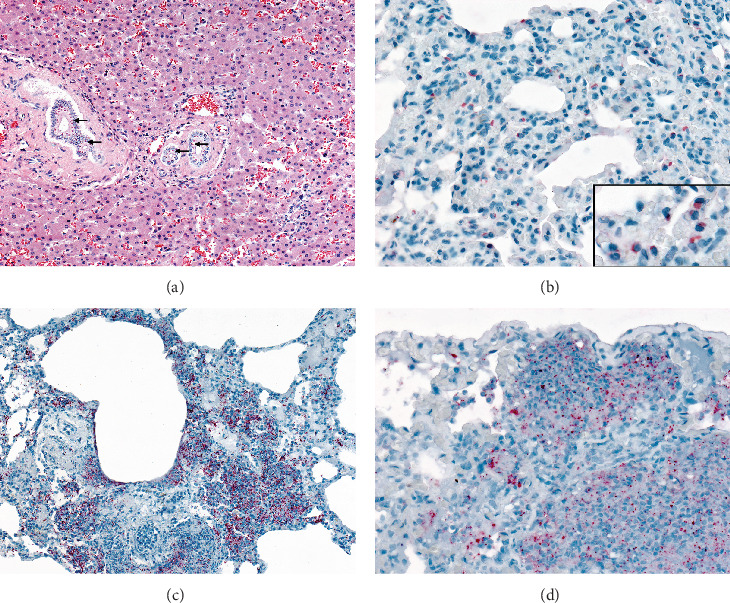
Histopathological and immunohistochemical findings of fatal CDV infection in free-ranging urbanized black-tufted marmosets. (A) Liver, NHP 1. Intracytoplasmic viral inclusions (arrows) within biliary duct epithelial cells (H&E, objective, 20 ×). (B) Lung, NHP 3. Immunostaining of CDV in pneumocytes (polymer-based IHC, objective, 40 ×). Inset. Cytoplasmatic immunostaining for CDV in pneumocytes. (C) Lung, NHP 2. Immunolabeling of *Bordetella* species within bronchopneumonia areas (polymer-based IHC, objective, 40×). (D) Lung, NHP 5. Intralesional immunolabeling of *Haemophilus* species within pneumonic areas (polymer-based IHC, objective, 40 ×).

Morbillivirus-characteristic eosinophilic intracytoplasmic inclusion bodies were found in epithelial cells of the lungs, tongue, skin, urinary bladder (Figure 2D), renal pelvis (urothelium), and hepatic bile ducts (Figure 3A). Additional findings included lymphoid depletion and necrosis, scattered syncytial cells in the spleen, and severe lymphoid depletion of lymph nodes (Table 1).

IHC for CDV evidenced cytoplasmic labeling in epithelial cells with inclusion bodies across all five marmosets, notably in the liver (hepatic bile ducts), gallbladder (epithelium), lungs (bronchiolar epithelium, pneumocytes) (Figure 3B), gastric epithelium, tongue epithelium, skin (epidermis), urothelium, and mucocutaneous-junction epithelium. Additionally, some lymphocytic and histiocytic-like cells also had positivity for CDV in the spleen and lymph nodes. No specific immunoreactivity was detected in the CNS, adrenal, and kidneys (except for urothelial positivity at the renal pelvis).

Bacterial coinfections were confirmed in four animals: NHP 2 had bacterial bronchopneumonia with Gram-negative coccobacilli; *Bordetella* spp. confirmed by IHC (Figure 3C) and PCR; NHP 3 evidenced polymicrobial bronchopneumonia with Gram-negative rods; IHC detected *E. coli*, *Staphylococcus* spp., and *Haemophilus* spp., with *Haemophilus* spp. confirmed by PCR. A cardiac microabscess also contained Gram-positive cocci and IHC-labeled *Staphylococcus* spp. NHP 4 presented with myocarditis associated with chain-forming, Gram-positive cocci and was PCR-confirmed to be *Streptococcus* spp.; NHP 5 developed a bronchopneumonia caused by *Haemophilus* spp., confirmed by IHC and PCR (Figure 3D).

**Figure 4:**
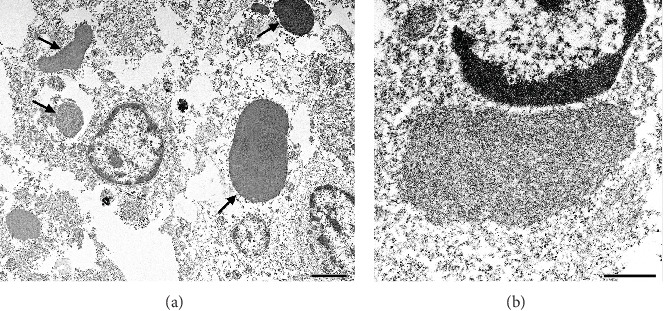
Transmission electron microscopy findings in the liver of NHP 3. (A) Intracytoplasmic viral inclusions (arrows) within hepatocytes, scale bar 2 µm. (B) Intracytoplasmic viral inclusion showing the structure of the viral nucleoprotein, scale bar 500 nm.

Numerous intracytoplasmic inclusion bodies in hepatocytes and bile duct epithelial cells, consistent with a paramyxovirus infection, were observed in the liver under TEM evaluation (Figure 4A). Typical viral nucleoprotein structures were demonstrated within the inclusions at higher magnification (Figure 4B).

### 3.3. Sequencing and Evolutionary Analyses

**Figure 5:**
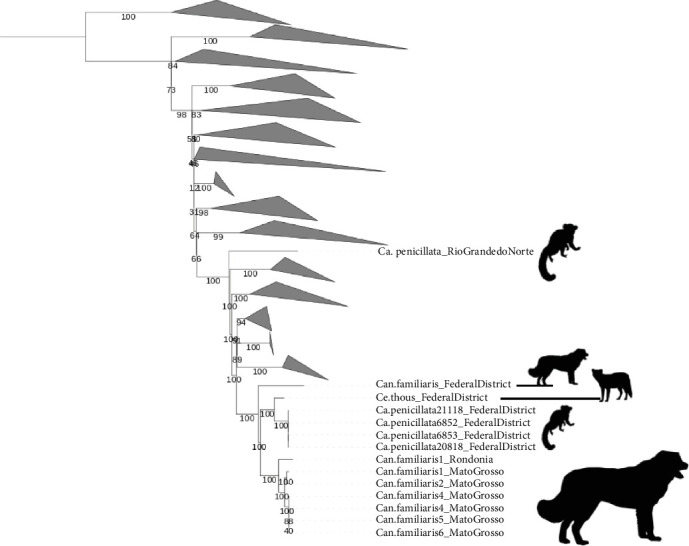
Maximum likelihood tree based on the H gene of CDV 665 variants depicting viruses from the present study and the most closely related sequences from a marmoset and dogs. Neighboring clades were collapsed for better visualization.

Initial sequencing, conducted for measles virus surveillance, demonstrated novel CDV genomes obtained from four marmosets (NHP 20018, NHP 21118, NHP 6852, and NHP 6853) and a crab-eating fox (*Cerdocyon thous*, CA584), all sampled in 2018 from the same geographical area. The phylogenetic analysis revealed marmoset-derived variants within a single clade, with the fox-derived virus serving as the sister group. This combined clade was, in turn, most closely related to dog-derived sequences from nearby states of Mato Grosso and Rondônia (GenBank: MT119972-77) (Figure 5). All of these sequences formed an ancestral clade, including the 2019 dog-derived sequence from the Federal District (MW460905) [22], suggesting a probable domestic dog origin with a subsequent spillover into local wildlife.

**Figure 6:**
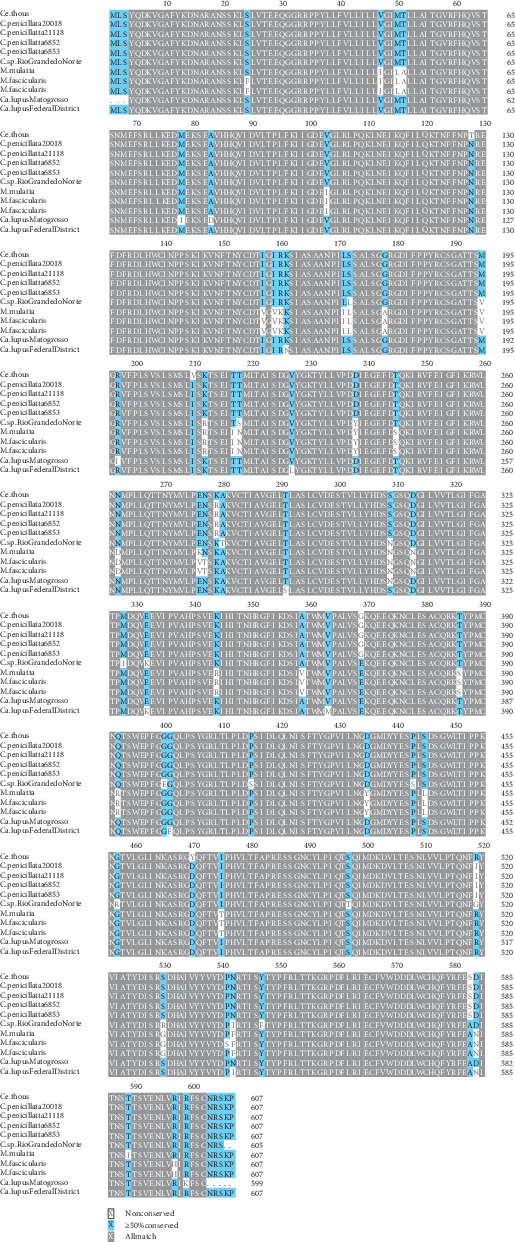
Multiple sequence alignment of H proteins from CDVs isolated from nonhuman primates. From top to bottom, the Genbank Accession Numbers for each sequence are: XCJ77521.1, XCJ77513.1, XCJ7729.1, XCJ7737.1, XCJ7744.1, XJQ60213.1, ADN86312.1, BAM15601.1, BAM15593.2, QQM99804.1, and QSV52435.1.

The multiple sequence alignment of the H protein had two substitutions (K279R and R519I) unique to the marmoset-derived viruses described in this study when compared to sequences from old world primates, domestic dogs, the fox [9, 25, 26], and the previously reported coinfected marmoset from Northwestern Brazil [10]. The latter strain exhibits its distinct substitutions (A280T, M328I, E331K, G400E, P415S, and P443S) (Figure 6), suggesting multiple independent adaptation events among CDV strains in marmosets. Additional unique amino acid changes were found in the present CDV variant: V proteins substitutions (S72Y and L90P, Supporting Information 1: S1), F proteins had R263K (Supporting Information 2: S2), and P proteins presented L90P and A426G (Supporting Information 3: S3).

**Figure 7:**
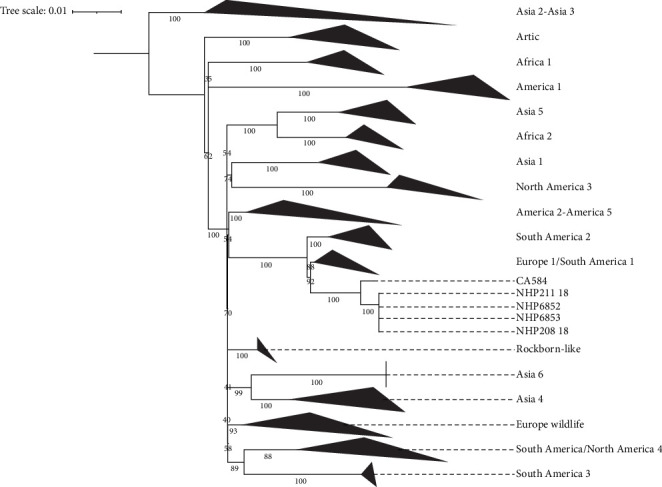
Maximum likelihood phylogeny of 98 CDV variants based on H genes.

Phylogenetic analysis of 98 H gene sequences placed the new variants as a sister clade to the Europe 1/South America 1 (ES1) lineage (Figure 7). In a broader phylogeny of 665 CDV H gene sequences, the marmoset and fox viruses clustered within the ES1 lineage (Figure 5). CDV strains differing by less than 5% in H gene sequences from known lineages are not considered distinct lineages [27]. Pairwise distance analysis showed that these variants fall below this threshold, supporting their inclusion in ES1 (Table 2).

## 4. Discussion

CDV is a globally distributed, potentially lethal pathogen in dogs and a threat to various wildlife species [28–31]. Like other highly pathogenic viruses, CDV spillover into free-ranging species can disrupt ecological balance [28, 32–34]. CDV infection in nonhuman primates (NHPs) remains rare, with only five outbreaks documented in captive old world NHPs from China and Japan over the past 30 years [9, 26, 27, 35–37]. These cases lacked clear epidemiological links, as affected animals had no reported contact with dogs.

In contrast, our study demonstrated interactions between unvaccinated domestic dogs and marmosets in urban environments, including squares, gardens, backyards, and even homes. Urbanized, free-ranging marmosets were observed consuming dog food, suggesting a novel route of infection via contaminated food or surfaces and implicating direct or indirect contact with canine secretions as a potential source of CDV transmission.

The respiratory and cutaneous signs observed in the marmosets suggest an acute systemic phase of CDV infection [32, 38]. Skin lesions in marmosets, primarily facial rashes, mirrored those observed in CDV-infected rhesus monkeys [9, 26, 27, 35–37]. Neurological signs, such as ataxia, stupor, blindness, and seizures observed in marmosets have also been reported in CDV infections in old world primates [27, 35, 36]. However, the marmosets in this study likely exhibited mainly acute-phase signs of CDV infection, which may reflect the influence of host factors, viral virulence, and immune status [32, 38], in contrast to the chronicity of infection and neurologic involvement.

Gross lesions were primarily observed in the skin, lungs, and lymphoid tissues, often in conjunction with bacterial coinfections. These findings are similar to previous CDV outbreaks in *Macaca mulatta*, where bronchopneumonia, skin rashes, and footpad thickening were prominent [9]. The pathological findings observed here also parallel those of measles in humans [39] and CDV infections in dogs and wildlife [40].

Histopathology predominantly showed epitheliotropic lesions across multiple organs. Notably, erosions and ulcers with eosinophilic intracytoplasmic inclusions affected the skin, mucocutaneous junctions, and tongue. In addition, pulmonary lesions included severe interstitial pneumonia with secondary bacterial bronchopneumonia, previously reported in monkeys with CDV [9]. Immunosuppression caused by CDV likely predisposed marmosets to coinfections, a pattern also seen in dogs [41]. Remarkably, we identified suppurative myocarditis possibly due to bacterial coinfection, a condition previously unreported in NHPs but also described in distemper-infected dogs [42].

Bacterial coinfections caused by *Streptococcus*, *Bordetella*, *E. coli*, *Staphylococcus*, and *Haemophilus* spp., likely contributed to the fatal outcomes. These bacteria are known opportunists in viral respiratory infections, including measles and respiratory syncytial virus [43], and have been documented in zoo-housed and facility-kept NHPs [1]. Their detection in free-ranging urban marmosets highlights the risk of pathogen spillover and spillback at the human–NHP interface, especially since these bacteria can colonize both species. Although no histological brain lesions were found in CDV-affected marmosets, neurological symptoms observed in some animals in this study may have resulted from indirect causes, such as hypoxia and cytokine release syndrome associated with a marked inflammatory response due to pulmonary damage and secondary bacterial infections.

Globally, phylogenetic studies reveal diverse CDV lineages across domestic and wild carnivores [29, 44, 45], with significant implications for both conservation and public health. Our phylogenetic analysis clustered the viral sequences within the Europe 1/South America 1 lineage, which is closely related to variants found in domestic dogs. This supports a likely spillover from dogs to *Cerdocyon thous* [44] and, in our findings, to marmosets. The urban setting of the cases further supports this ecological pathway. Importantly, these variants were phylogenetically distinct from vaccine strains, indicating that the outbreak was caused by circulating field strains rather than vaccine reversion [29].

The ability of CDV and MV to infect multiple hosts is linked to interactions between the viral H protein and the signaling lymphocyte activation molecule (SLAM) [38, 46]. CDV-H preferentially binds to canine SLAM, while MV-H can bind to human, tamarin, and dog SLAM [38, 46]. In our study, the marmoset-infecting viruses presented an R519I substitution in the H protein, previously associated with enhanced binding to noncanid SLAM receptors in lions and hyenas [46], while retaining an aspartic acid at position 540. Key amino acids related to receptor binding for CDV are located in the H protein at positions 519 and 540 [30]. These substitutions may facilitate the infection of platyrrhine primates, but the absence of known substitutions (D540G and M548T) suggests that the marmoset viruses would not be able to infect humans [47]. However, CDV's ability to bind to human Nectin-4 and replicate in Vero cells expressing primate SLAM receptors remains concerning for its potential to cause cross-species infection [37].

Despite speculation linking CDV to Paget's disease and multiple sclerosis, there is no confirmed evidence of human infection [48]. Encouragingly, studies in MV-vaccinated macaques demonstrated reduced CDV replication and partial cross-protection [11], while human serum had cross-neutralizing activity [49]. MV vaccination may, therefore, offer incidental protection against potential CDV spillover.

Given the cutaneous lesions and the surge in measles cases in Brazil in 2019, MV was initially suspected in these marmosets. However, molecular and metagenomic analyses confirmed the presence of CDV. The decline in global MV vaccination rates may create ecological opportunities for animal morbilliviruses, such as CDV to infect humans [50]. Therefore, monitoring CDV's zoonotic potential is essential, especially in the context of expanding human-wildlife interfaces.

To date, only five natural CDV infections in NHPs have been documented, with four occurring in Asia and one in the Americas [10, 29]. Our study reports a novel outbreak in free-ranging primates in Brazil. Notably, CDV was the sole viral pathogen detected, suggesting it can infect immunocompetent marmosets. Comparison of H protein sequences evidenced genetic differences between the viruses in our study and those reported in Brazil [10], which belong to different lineages and were detected in geographically distant regions (Midwest vs. northwest Brazil]. This suggests that independent spillover events and distinct evolutionary trajectories pose a risk to vulnerable primate populations.

Some limitations of this study included the difficulty in investigating the epidemiological context of the outbreak and the inability to assess additional factors that may have influenced the onset, progression, and occurrence of the disease in free-ranging marmosets. Furthermore, the current understanding of CDV infection in Neotropical primates remains scarce, which limits meaningful comparisons and complicates the interpretation of our findings, particularly the absence of pathological lesions in the CNS and the lack of viral detection by IHC.

## 5. Conclusions

While human urbanization contributed to the emergence of measles, ongoing urban expansion and associated land-use changes now pose new risks to Neotropical primates of exposure to other morbilliviruses, including CDV. Our findings underscore the urgent need for coordinated, multidisciplinary strategies to mitigate CDV transmission at the interface between humans, domestic animals, and wildlife. These strategies should include robust vaccination campaigns, control of stray and free-ranging domestic animals, and active disease surveillance.

Currently, there is no global program framework for systematically monitoring infectious diseases in wild NHPs. In Brazil, the national YF surveillance program, although primarily aimed at preventing human infections, has proven effective in detecting other zoonotic threats, such as this CDV outbreak, underscoring its value in identifying spillover events with both conservation and public health implications. Strengthening and expanding such integrated surveillance systems is critical to advancing a One Health approach that safeguards human health, animal populations, and ecosystem stability.

## Figures and Tables

**Table 1:** Clinical and pathological features of natural, fatal CDV infections in free-ranging black-tufted marmosets.

NHP	Clinical presentation and evolution	Pathological features	
		Gross	Histopathology
1	Lethargy and ataxia; died without treatment	Skin: scabs around the nose and ulcers on the upper lip Lungs: heavy and diffusely reddish Spleen: severe enlargement	Skin: multifocal areas of erosion, ulceration, forming vesicles Tongue: multifocal areas of erosion, forming vesicles, mild multifocal lymphoplasmacytic glossitis Lungs: interstitial pneumonia and mild bronchiolar necrosis with syncytial cells Spleen: white pulp necrosis Inclusion bodies: hepatic bile ducts, gastric, tongue, skin, bronchiolar, gallbladder, renal pelvis, and urinary bladder epithelium

2	Stupor, amaurosis, seizures, ulcers, and vesicles on the facial skin and tongue, dying within 1 day despite treatment	Skin: severe rashes, ulcers, and thickening of the nasal skin, periorbital area, and oral mucocutaneous junction Tongue: erosion and white plaques on the ventral and dorsal surface Lungs: heavy, firm, wet, partially collapsed with dark red mottled areas of predominately cranioventral consolidation Spleen: severe enlargement	Skin: multifocal areas of erosion, ballooning degeneration Tongue: multifocal areas of erosion, hyperkeratosis, mild multifocal lymphoplasmacytic glossitis, and ballooning degeneration Lungs: suppurative bronchopneumonia, edema, interstitial pneumonia with syncytial cells, and bronchiolar necrosis. Spleen: white pulp necrosis. Inclusion bodies: hepatic bile ducts, renal pelvis, tongue, skin urinary bladder and mucocutaneous boundary epithelium

3	Lethargy; lesions on the facial skin and tongue similar to NHP 2, and died during clinical evaluation	Skin: severe rashes, ulcers, and scabs on the nose, periorbital area, and oral mucocutaneous junction Tongue: discrete multifocal erosion and white plaques on the ventral and dorsal surface Lungs: heavy, firm, wet, partially collapsed with dark red mottled areas of predominately cranioventral consolidation Spleen and submandibular lymph nodes: severe enlargement	Skin: multifocal areas of erosion, focally extensive ulcerative dermatitis, scabs, and hyperkeratosis Mucocutaneous boundary: scabs, hyperkeratosis Tongue: multifocal areas of erosion, hyperkeratosis, mild multifocal lymphoplasmacytic glossitis, and ballooning degeneration Lungs: suppurative bronchopneumonia, interstitial pneumonia with syncytial cells, and bronchiolar necrosis Heart: microabscess with myriad bacteria Spleen: white pulp necrosis Lymph nodes: lymphoid depletion and rare syncytial cells Inclusion bodies: hepatic bile ducts, renal pelvis, urinary bladder, skin, mucocutaneous boundary, tongue bronchiolar and endometrial epithelium

4	Lethargy and ataxia; died without treatment	Skin: severe rashes, ulcers, and scabs on the nose, periorbital area, and mouth mucocutaneous junction Tongue: focally extensive erosion. Lungs: heavy, firm, wet, partially collapsed with dark red mottled areas of predominately cranioventral consolidation Spleen: severe enlargement	Skin: multifocal areas of erosion, focally extensive ulcerative dermatitis, ballooning degeneration, hyperkeratosis Mucocutaneous boundary: scabs, hyperkeratosis Tongue: epidermal necrosis, hyperkeratosis, multifocal lymphoplasmacytic and neutrophilic glossitis, and ballooning degeneration Lungs: suppurative bronchopneumonia, interstitial pneumonia with syncytial cells, and bronchiolar necrosis Heart: suppurative myocarditis with myriad bacteria Spleen: white pulp necrosis Inclusion bodies: hepatic bile ducts, renal pelvis, urinary bladder skin, mucocutaneous boundary, tongue bronchiolar, granulosa cells and endometrial epithelium

5	Lethargy and facial skin lesions; died within 3 days without treatment	Skin: rashes mainly around oral mucocutaneous junction. Tongue: erosion and white plaques on the dorsal surface Lungs: dark red mottled areas of predominately cranioventral consolidation Spleen: severe enlargement	Skin and mucocutaneous boundary: multifocal areas of erosion, ulceration, pustules, scabs and ballooning degeneration Tongue: multifocal areas of erosion, pustules, hyperkeratosis, mild multifocal lymphoplasmacytic glossitis and ballooning degeneration Lungs: suppurative bronchopneumonia, edema with syncytial cells and bronchiolar necrosis Spleen: white pulp necrosis Inclusion bodies: hepatic bile ducts, renal pelvis, urinary bladder skin, mucocutaneous boundary, tongue bronchiolar and endometrial epithelium

**Table 2:** Similarity distance matrix of H gene between representative CDV lineages.

GenBank accession number	Z77672.1 (%)	DQ226087.1 (%)	DQ903854.1 (%)	EU716337.1 (%)	EU743934.1 (%)	EU716075.1 (%)	FJ392652.1 (%)	FJ461695.1 (%)	GU266280.1 (%)	JN153020.1 (%)	JF283477.1 (%)	JN812976.1 (%)	KJ437596.1(%)	KT266736.1 (%)	MF964178.1 (%)	MK617349.1 (%)	MW535267,1 (%)	CA584 (%)	PNH208_18 (%)
Z77672.1	0.00	5.32	8.06	3.25	10.64	6.47	2.36	5.15	2.58	4.28	4.27	4.44	5.37	6.41	5.48	4.50	5.56	2.08	2.19
DQ226087.1	5.32	0.00	8.44	4.93	10.64	6.74	5.43	5.37	3.95	5.81	6.13	6.30	6.91	8.06	7.07	5.87	7.24	5.70	5.92
DQ903854.1	8.06	8.44	0.00	8.00	13.32	9.38	8.33	8.33	7.13	8.77	9.23	8.77	9.21	10.58	9.65	8.83	9.92	8.44	8.55
EU716337.1	3.25	4.93	8.00	0.00	10.20	6.20	3.62	5.04	2.31	4.11	3.34	4.50	5.10	6.20	5.26	4.33	4.44	3.84	3.84
EU743934.1	10.64	10.64	13.32	10.20	0.00	5.70	10.75	10.58	9.38	11.24	11.83	11.40	11.90	12.45	12.23	11.29	11.79	10.80	10.91
EU716075.1	6.47	6.74	9.38	6.20	5.70	0.00	6.74	6.74	5.15	7.24	7.37	7.57	8.00	8.72	8.28	7.24	7.95	6.91	7.07
FJ392652.1	2.36	5.43	8.33	3.62	10.75	6.74	0.00	5.37	2.96	4.99	4.52	5.10	5.65	6.74	5.54	5.15	5.98	2.80	2.96
FJ461695.1	5.15	5.37	8.33	5.04	10.58	6.74	5.37	0.00	4.06	5.81	6.13	6.09	6.80	8.17	7.13	5.98	6.74	5.43	5.59
GU266280.1	2.58	3.95	7.13	2.31	9.38	5.15	2.96	4.06	0.00	3.18	3.16	3.40	4.11	5.26	4.55	3.45	4.42	3.13	3.23
JN153020.1	4.28	5.81	8.77	4.11	11.24	7.24	4.99	5.81	3.18	0.00	4.77	5.43	5.48	7.02	6.36	4.99	6.09	4.77	4.88
JF283477.1	4.27	6.13	9.23	3.34	11.83	7.37	4.52	6.13	3.16	4.77	0.00	5.02	5.88	6.44	5.88	5.70	5.94	4.71	4.71
JN812976.1	4.44	6.30	8.77	4.50	11.40	7.57	5.10	6.09	3.40	5.43	5.02	0.00	6.52	7.29	4.55	5.76	6.47	5.21	5.32
KJ437596.1	5.37	6.91	9.21	5.10	11.90	8.00	5.65	6.80	4.11	5.48	5.88	6.52	0.00	7.35	7.40	5.87	6.30	5.76	5.87
KT266736.1	6.41	8.06	10.58	6.20	12.45	8.72	6.74	8.17	5.26	7.02	6.44	7.29	7.35	0.00	7.95	6.63	7.68	7.07	7.02
MF964178.1	5.48	7.07	9.65	5.26	12.23	8.28	5.54	7.13	4.55	6.36	5.88	4.55	7.40	7.95	0.00	6.58	7.13	5.98	6.03
MK617349.1	4.50	5.87	8.83	4.33	11.29	7.24	5.15	5.98	3.45	4.99	5.70	5.76	5.87	6.63	6.58	0.00	6.63	5.32	5.43
MW535267.1	5.56	7.24	9.92	4.44	11.79	7.95	5.98	6.74	4.42	6.09	5.94	6.47	6.30	7.68	7.13	6.63	0.00	6.14	6.25
CAS584	2.08	5.70	8.44	3.84	10.80	6.91	2.80	5.43	3.13	4.77	4.71	5.21	5.76	7.07	5.98	5.32	6.14	0.00	0.66
NHP 208_18	2.19	5.92	8.55	3.84	10.91	7.07	2.96	5.59	3.23	4.88	4.71	5.32	5.87	7.02	6.03	5.43	6.25	0.66	0.00

*Note*: Z77672.1 (Germany,Europe-1/South America-1 lineage); DQ226087.1 (Italy,Artic lineage); DQ903854.1 (Hungary,America-1 lineage); EU716337.1 (USA,America-2 lineage); EU743934.1 (China,Asia-3 lineage); EU716075.1 (South Korea,Asia-1 lineage); FJ392652.1 (Argentina,South America-2); FJ461695.1 (South Africa,Africa-1 lineage); GU266280.1 (Denmark,Rockborn-like lineage); JN153020.1 (Germany,Europe wildlife lineage); JF283477.1 (USA,America-5 lineage); JN812976.1 (Tanzania,Africa-2 lineage); KJ437596.1 (China,Asia-4 lineage); KT266736.1 (Mexico,North America-3 lineage); MF964178.1 (India,Asia-5 lineage); MK617349.1 (Colombia,South America/North America-4 lineage); MW535267.1 (China,Asia-6 lineage); CAS584 (Ce.thous584); NHP 208_18 (Ca.penicillata20818).

## Data Availability

All the genome sequences in this study were deposited in GenBank (Accession Numbers: PP847352, PP847351, PP847348, PP847350, and PP847349).

## Supporting Information

Supporting Information 1 S1. Multiple sequence alignment between V proteins encoded from nonhuman primate-derived and canid-derived CDV isolates. From top to bottom, the Genbank Accession Numbers for each sequence are: XCJ77523.1, XCJ77515.1, XCJ77531.1, XCJ77539.1, XCJ77547.1, BAM15589.1, and BAM15597.2. Sequences for M.mulata and Canis lupus were translated from HM852904 and AB753775, respectively.

Supporting Information 2 S2. Multiple sequence alignment between F proteins encoded from nonhuman primate-derived and canid-derived CDV isolates. From top to bottom, the Genbank Accession Numbers for each sequence are: XCJ77520.1, XCJ77512.1, XCJ77528.1, XCJ77536.1, XCJ77544.1, XJQ60212.1, ADN86311.1, BAM15600.1, BAM15592.2, and QSV52434.1.

Supporting Information 3 S3. Multiple sequence alignment between P encoded from nonhuman primate-derived and canid-derived CDV isolates. From top to bottom, the Genbank Accession Numbers for each sequence are: XCJ77517.1, XCJ77509.1, XCJ77525.1, XCJ77533.1, XCJ77542.1, XJQ60209.1, ADN86309.1, BAM15596.1, BAM15588.2, and QSV52432.1.
