# Supplementary material for: Emergence of a Novel Canine Distemper Virus Variant in Urbanized Free-Ranging Marmosets (Callithrix penicillata)
Source: Transbound Emerg Dis. 2025 Aug 15;2025:4818076. doi: 10.1155/tbed/4818076 (PMC12373474; doi:10.1155/tbed/4818076)
Supplement: Supporting Information 1 — S1. Multiple sequence alignment between V proteins encoded from nonhuman primate-derived and canid-derived CDV isolates. From top to bottom, the Genbank Accession Numbers for each sequence are: XCJ77523.1, XCJ77515.1, XCJ77531.1, XCJ77539.1, XCJ77547.1, BAM15589.1, and BAM15597.2. Sequences for M.mulata and Canis lupus were translated from HM852904 and AB753775, respectively. [file 4818076.f1.pdf]

```
                                     10        20        30        40        50        60        70
Ce.thous              MAEEQAYHVSKGLECLKALRENPPDLEEIQEVSSIRDQTRNPGQENGTAGMQEEEVSQDLDESHEPAKGS  70
C.penicillata20018    MAEEQAYHVSKGLECLKALRENPPDLEEIQEVSSIRDQTRNPGQENGTAGMQEEEVSQDLDESHEPAKGS  70
C.penicillatta21118   MAEEQAYHVSKGLECLKALRENPPDLEEIQEVSSIRDQTRNPGQENGTAGMQEEEVSQDLDESHEPAKGS  70
C.penicillatta6852    MAEEQAYHVSKGLECLKALRENPPDLEEIQEVSSIRDQTRNPGQENGTAGMQEEEVSQDLDESHEPAKGS  70
C.penicillatta6853    MAEEQAYHVSKGLECLKALRENPPDLEEIQEVSSIRDQTRNPGQENGTAGMQEEEVSQDLDESHEPAKGS  70
M.mulatta             MAEEQAYHVNKGLECLKALRENPPDIEEIQEVSSIRDQTRNPGQENGTASMQEEEVSQDLDESHEPAKGS  70
M.fascicularis        MAEEQAYHVNKGLECLKALRENPPDIEEIQEVSSIRDQTRNPGQENGTASMQEEEVSQDLDESHEPTKGS  70
M.fascicularis        MAEEQAYHVNKGLECLKALRENPPDIEEIQEVSSIRDQTRNPGQENGTASMQEEEVSQDLDESHEPTKGS  70
Ca.lupus old world    MAEEQAYHVNKGLECLKALRENPPDIEEIQEVSSIRDQTRNPGQEDGTASKQEEEVSQDLDESHEPAKGS  70

                               80        90        100       110       120       130       140
Ce.thous              NSVGHVLQNNPGSGKGNTALVEAEQPAKDVIQPGPGIRCYHVYDHSGEEVKGIEDADSLVVPAGAVSNRG  140
C.penicillata20018    NYVGHVLQNNPGSGKGNTAPVEAEQPAKDVIQPGPGIRCYHVYDHSGEEVKGIEDADSLVVPAGAVSNRG  140
C.penicillatta21118   NYVGHVLQNNPGSGKGNTAPVEAEQPAKDVIQPGPGIRCYHVYDHSGEEVKGIEDADSLVVPAGAVSNRG  140
C.penicillatta6852    NYVGHVLQNNPGSGKGNTAPVEAEQPAKDVIQPGPGIRCYHVYDHSGEEVKGIEDADSLVVPAGAVSNRG  140
C.penicillatta6853    NYVGHVLQNNPGSGKGNTAPVEAEQPAKDVIQPGPGIRCYHVYDHSGEEVKGIEDADSLVVPAGAVSNRG  140
M.mulatta             NYLGHVLQNNPGCGESNTALVEAEQPAKDDIQPGPGIRCYHVYDHSGEEVKGIEDADSLVVPAGAVSNRG  140
M.fascicularis        NYLGHVLQNNPGCGESNTALVEAEQPAKDDIQPGPGIRCYHVYDHSGEEVKGIEDADSLVVPAGAVSNRG  140
M.fascicularis        NYLGHVLQNNPGCGESNTALVEAEQPAKDDIQPGPGIRCYHVYDHSGEEVKGIEDADSLVVPAGAVSNRG  140
Ca.lupus old world    NYVGHVLQNNPGCGESNTALVEAEQPAKDDVQPGPGIRCYHVYDHSGEEVKGIEDADSLVVPVGAVSNRG  140

                             150       160       170       180       190       200       210
Ce.thous              FERGEGSLDDSTEDSGEDYSEGNASSNWGYSFGLKPDRAADVSMLMEEELSALLKTSRNVGIQKRDGKTL  210
C.penicillata20018    FERGEGSLDDSTEDSGEDYSEGNASSNWGYSFGLKPDRAADVSMLMEEELSALLKTSRNVGIQKRDGKTL  210
C.penicillatta21118   FERGEGSLDDSTEDSGEDYSEGNASSNWGYSFGLKPDRAADVSMLMEEELSALLKTSRNVGIQKRDGKTL  210
C.penicillatta6852    FERGEGSLDDSTEDSGEDYSEGNASSNWGYSFGLKPDRAADVSMLMEEELSALLKTSRNVGIQKRDGKTL  210
C.penicillatta6853    FERGEGSLDDSTEDSGEDYSEGNASSNWGYSFGLKPDRAADVSMLMEEELSALLKTSRNVGIQKRDGKTL  210
M.mulatta             FERGEGSPDDSTEDSGEDYSEGNASSNWGYSFGLKPDRAADVSMLMEEELSALLKTSRNVGIKKRDGKTL  210
M.fascicularis        FERGEGSPDDSTEDSGEDYSEGNASSNWGYSFGLKPDRAADVSMLMEEELSALLKTSRNVGIKKRDGKTL  210
M.fascicularis        FERGEGSPDDSTEDSGEDYSEGNASSNWGYSFGLKPDRAADVSMLMEEELSALLKTSRNVGIKKRDGKTL  210
Ca.lupus old world    FERGEGSLDDSTEDSGEDYSEGNASSNWGYSFGLKPDRAADVSMLMEEELSALLKTSRNVGIKKRDGMTL  210

                             220       230       240       250       260       270       280
Ce.thous              QFPHNPEGKIGDPECGSIKKGTGERSASHGMEIVAGSTNGATQSALKSTGGSSGPSVSAENVRQPAVSAK  280
C.penicillata20018    QFPHNPEGKIGDPECGSIKKGTGERSASHGMEIVAGSTNGATQSALKSTGGSSGPSVSAENVRQPAVSAK  280
C.penicillatta21118   QFPHNPEGKIGDPECGSIKKGTGERSASHGMEIVAGSTNGATQSALKSTGGSSGPSVSAENVRQPAVSAK  280
C.penicillatta6852    QFPHNPEGKIGDPECGSIKKGTGERSASHGMEIVAGSTNGATQSALKSTGGSSGPSVSAENVRQPAVSAK  280
C.penicillatta6853    QFPHNPEGKIGDPECGSIKKGTGERSASHGMEIVAGSTNGATQSALKSTGGSSGPSVSAENVRQPAVSAK  280
M.mulatta             QFPHNPEGKTEDPECGSIKKGTGERSASHGMGIVAGSTNGATQSALKSTGGSSGPSVSAENVRQPAMNAK  280
M.fascicularis        QFPHNPEGKTEDPECGSIKKGTGERSASHGMGIVAGSTNGATQSALKSTGGSSGPSVSAENVRQPAMSAK  280
M.fascicularis        QFPHNPEGKTEDPECGSIKKGTGERSASHGMGIVAGSTNGATQSALKSTGGSSGPSVSAENVRQPAMSAK  280
Ca.lupus old world    QFPHNPEGKTEDPECGSIKKGTGERSASHGMGIVAGSTNGATQSALKSTGGSSGPSVSAENVRQPAMNAK  280

                             290
Ce.thous              MTQKCKPESGTQLPPKTSN  299
C.penicillata20018    MTQKCKPESGTQLPPKTSN  299
C.penicillatta21118   MTQKCKPESGTQLPPKTSN  299
C.penicillatta6852    MTQKCKPESGTQLPPKTSN  299
C.penicillatta6853    MTQKCKPESGTQLPPKTSN  299
M.mulatta             MTQKCKLESGMQLPPKTSN  299
M.fascicularis        MTQKCKPESGMQLPPKTSN  299
M.fascicularis        MTQKCKPESGMQLPPKTSN  299
Ca.lupus old world    MTQKCKPESGTQLPPRTSN  299
```

X non-conserved

X $\geq$ 50% conserved

X all match
