# Supplementary material for: Emergence of a Novel Canine Distemper Virus Variant in Urbanized Free-Ranging Marmosets (Callithrix penicillata)
Source: Transbound Emerg Dis. 2025 Aug 15;2025:4818076. doi: 10.1155/tbed/4818076 (PMC12373474; doi:10.1155/tbed/4818076)
Supplement: Supporting Information 2 — S2. Multiple sequence alignment between F proteins encoded from nonhuman primate-derived and canid-derived CDV isolates. From top to bottom, the Genbank Accession Numbers for each sequence are: XCJ77520.1, XCJ77512.1, XCJ77528.1, XCJ77536.1, XCJ77544.1, XJQ60212.1, ADN86311.1, BAM15600.1, BAM15592.2, and QSV52434.1. [file 4818076.f2.pdf]

```
                                     10        20        30        40        50        60
Ce.thous                    MHNKIPKGSRTPTPTQQDLPKQYSIKSAETKTSQARHSITSAQRSTRHGPRTSDRPVHYIMNKTR  65
C.penicillata20018          MHNKIPKGSKTPTPTQQDLPQQYSIKSAETKTSQARHSITSAQRSTRHGPRTSDRPVHYIMNKTR  65
C.penicillatta21118         MHNKIPKGSKTPTPTQQDLPQQYSIKSAETKTSQARHSITSAQRSTRHGPRTSDRPVHYIMNKTR  65
C.penicillatta6852          MHNKIPKGSKTPTPTQQDLPQQYSIKSAETKTSQARHSITSAQRSTRHGPRTSDRPVHYIMNKTR  65
C.penicillatta6853          MHNKIPKGSKTPTPTQQDLPQQYSIKSAETKTSQARHSITSAQRSTRHGPRTSDRPVHYIMNKTR  65
C.sp. Rio Grande do Norte   ............................................................MNRTR  5
M.mulatta                   MHNKTPKKSKPLPHTRQNPLQQHSTKSAETKTSQGRYSITSAQRSTHHGPRTSNRPVHYIMNRTR  65
M.fascicularis              MHNKTPKKSKPLPHTRQNPLQQHSTKSAETKTSQGRYSITSAQRSTHHGPRTSNRPVHYIMNRTR  65
M.fascicularis              MHNKTPKKSKPLPHTRQNPLQQHSTKSAETKTSQGRYSITSAQRSTHHGPRTSNRPVHYIMNRTR  65
Ca.lupus Federal District   MHNKIPKGSKTLTPTQQDLPQRHNIKSAETRTSPARHSTTSAQRSTHHGPRTSDRPVHYIMNRTR  65

                                 70        80        90        100       110       120       130
Ce.thous                    SRKQAGYRLDNILAHGDHEGIIHHTPGSVSQGVRSRFKRQQSNATSSGSQCTWLVLWCIGMASLF  130
C.penicillata20018          SRKQAGYRLDNILAHGDHEGIIHHTPGSVSQGVRSRFKRQQSNATCSGSQCTWLVLWCIGMASLF  130
C.penicillatta21118         SRKQAGYRLDNILAHGDHEGIIHHTPGSVSQGVRSRFKRQQSNATCSGSQCTWLVLWCIGMASLF  130
C.penicillatta6852          SRKQAGYRLDNILAHGDHEGIIHHTPGSVSQGVRSRFKRQQSNATCSGSQCTWLVLWCIGMASLF  130
C.penicillatta6853          SRKQAGYRLDNILAHGDHEGIIHHTPGSVSQGVRSRFKRQQSNATCSGSQCTWLVLWCIGMASLF  130
C.sp. Rio Grande do Norte   SCKPASYRSDNIPAHGDHEGIAHHTPESVSQGVRSRFKRRQSSATSSGFQCTWLVLWCIGIASLV  70
M.mulatta                   SCMQTSHRSDNILDHRDHEGIIHHTPESVTQGVGFWFKRRRSNATNAGSQCTWLVLWCIGIASLF  130
M.fascicularis              SCMQTSHRSDNIPDHRDHEGIIHHTPESVTQGVGFWFKRRRSNATNAGSQCTWLVLWCIGIASLF  130
M.fascicularis              SCMQTSHRSDNIPDHRDHEGIIHHTPESVTQGVGFWFKRRRSNATNAGSQCTWLVLWCIGIASLF  130
Ca.lupus Federal District   SCKQAGYRSDNIPAHGDHEGIIHHTPESVSQGVRSRFKRQQSNATSSGSQCTWLVLWCIGIASLF  130

                                       140       150       160       170       180       190
Ce.thous                    LCSKAQIHWNNLSTIGIIGTDSVHYKIMTRPSHQYLVIKLMPNVSLIDNCTKVELGEYEKLLNSV  195
C.penicillata20018          LCSKAQIHWNNLSTIGIIGTDSVHYKIMTRPSHQYLVIKLMPNVSLIDNCTKAELGEYEKLLNSV  195
C.penicillatta21118         LCSKAQIHWNNLSTIGIIGTDSVHYKIMTRPSHQYLVIKLMPNVSLIDNCTKAELGEYEKLLNSV  195
C.penicillatta6852          LCSKAQIHWNNLSTIGIIGTDSVHYKIMTRPSHQYLVIKLMPNVSLIDNCTKAELGEYEKLLNSV  195
C.penicillatta6853          LCSKAQIHWNNLSTIGIIGTDSVHYKIMTRPSHQYLVIKLMPNVSLIDNCTKAELGEYEKLLNSV  195
C.sp. Rio Grande do Norte   LCSEAQIHWNNLSTIGIIGTDSVHYKIMTRPSHQYLVIKLMPNVSLIDNCTKAELGEYEKLLSSV  135
M.mulatta                   LCSKAQIHWNNLSTIGIIGTDSVHYKIMTRPSHQYLVIKLMPNVSLIDNCTKAELGEYEKLLNSV  195
M.fascicularis              LCSKAQIHWNNLSTIGIIGTDSVHYKIMTRPSHQYLVIKLMPNVSLIDNCTKAELGEYEKLLNSV  195
M.fascicularis              LCSKAQIHWNNLSTIGIIGTDSVHYKIMTRPSHQYLVIKLMPNVSLIDNCTKAELGEYEKLLNSV  195
Ca.lupus Federal District   LCSKAQIHWNNLSTIGIIGTDSVHYKIMTRPSHQYLVIKLMPNVSLIDNCTKAELGEYEKLLSSV  195

                                200       210       220       230       240       250       260
Ce.thous                    LEPINQALTLMTKNVKPLQSVGSGRRQKRFAGVVLAGAALGVATAAQITAGIALHQSNLNAQAIQ  260
C.penicillata20018          LEPINQALTLMTKNVKPLQSVGSGRRQKRFAGVVLAGAALGVATAAQITAGIALHQSNLNAQAIQ  260
C.penicillatta21118         LEPINQALTLMTKNVKPLQSVGSGRRQKRFAGVVLAGAALGVATAAQITAGIALHQSNLNAQAIQ  260
C.penicillatta6852          LEPINQALTLMTKNVKPLQSVGSGRRQKRFAGVVLAGAALGVATAAQITAGIALHQSNLNAQAIQ  260
C.penicillatta6853          LEPINQALTLMTKNVKPLQSVGSGRRQKRFAGVVLAGAALGVATAAQITAGIALHQSNLNAQAIQ  260
C.sp. Rio Grande do Norte   LEPINQALTLMTKNVKPLQSVGSGRRQRRFAGVVLAGAALGVATAAQITAGIALHQSNLNAQAIQ  200
M.mulatta                   LEPINQALTLMTNNVKLLQSVGSGRRQRRFAGVVLAGAALGVATAAQITAGIALHQSNLNAQAIQ  260
M.fascicularis              LEPINQALTLMTNNVKLLQSVGSGRRQRRFAGVVLAGAALGVATAAQITAGIALHQSNLNAQAIQ  260
M.fascicularis              LEPINQALTLMTNNVKLLQSVGSGRRQRRFAGVVLAGAALGVATAAQITAGIALHQSNLNAQAIQ  260
Ca.lupus Federal District   LEPINQALTLMTRNVKPLQSVGSGRRQRRFAGVVLAGAALGVATAAQITAGIALHQSNLNAQAIQ  260

                                      270       280       290       300       310       320
Ce.thous                    SLRTSLEQSNKAIEEIREATQETIIAVQGVQDYVNNELVPAMQHMSCELVGQRLGLKLLRYYTEL  325
C.penicillata20018          SLKTSLEQSNKAIEEIREATQETIIAVQGVQDYVNNELVPAMQHMSCELVGQRLGLKLLRYYTEL  325
C.penicillatta21118         SLKTSLEQSNKAIEEIREATQETIIAVQGVQDYVNNELVPAMQHMSCELVGQRLGLKLLRYYTEL  325
C.penicillatta6852          SLKTSLEQSNKAIEEIREATQETIIAVQGVQDYVNNELVPAMQHMSCELVGQRLGLKLLRYYTEL  325
C.penicillatta6853          SLKTSLEQSNKAIEEIREATQETIIAVQGVQDYVNNELVPAMQHMSCELVGQRLGLKLLRYYTEL  325
C.sp. Rio Grande do Norte   SLRTSLEQSNKAIEEIREATQETIIAVQGVQDYVNNELVPAMQHMSCELVGQRLGLKLLRYYTEL  265
M.mulatta                   SLRTSLEQSNKAIEEIREATQETVIAVQGVQDYVNNELVPAMQHMSCELVGQRLGLKLLRYYTEL  325
M.fascicularis              SLRTSLEQSNKAIEEIREATQETVIAVQGVQDYVNNELVPAMQHMSCELVGQRLGLKLLRYYTEL  325
M.fascicularis              SLRTSLEQSNKAIEEIREATQETVIAVQGVQDYVNNELVPAMQHMSCELVGQRLGLKLLRYYTEL  325
Ca.lupus Federal District   SLRTSLEQSNKAIEEIREATQETIIAVQGVQDYVNNELVPAMQHMSCELVGQRLGLKLLRYYTEL  325

                                    330       340       350       360       370       380       390
Ce.thous                    LSIFGPSLRDPISAEISIQALSYALGGEIHKILEKLGYSGNDMIAILESRGIKTKITHVDIPGKL  390
C.penicillata20018          LSIFGPSLRDPISAEISIQALSYALGGEIHKILEKLGYSGNDMIAILESRGIKTKITHVDIPGKL  390
C.penicillatta21118         LSIFGPSLRDPISAEISIQALSYALGGEIHKILEKLGYSGNDMIAILESRGIKTKITHVDIPGKL  390
C.penicillatta6852          LSIFGPSLRDPISAEISIQALSYALGGEIHKILEKLGYSGNDMIAILESRGIKTKITHVDIPGKL  390
C.penicillatta6853          LSIFGPSLRDPISAEISIQALSYALGGEIHKILEKLGYSGNDMIAILESRGIKTKITHVDIPGKL  390
C.sp. Rio Grande do Norte   LSVFGPSLRDPISAEISIQALSYALGGEIHKILEKLGYSGNDMIAILESRGIKTKITHVDLPGKL  330
M.mulatta                   LSIFGPSLRDPISAEISIQALSYALGGEIHKILEKLGYSGNDMIAILESRGIKTKITHVDLPGKL  390
M.fascicularis              LSIFGPSLRDPISAEISIQALSYALGGEIHKILEKLGYSGNDMIAILESRGIKTKITHVDLPGKL  390
M.fascicularis              LSIFGPSLRDPISAEISIQALSYALGGEIHKILEKLGYSGNDMIAILESRGIKTKITHVDLPGKL  390
Ca.lupus Federal District   LSIFGPSLRDPISAEISIQALSYALGGEIHKILEKLGYSGNDMIAILESRGIKTKITHVDIPGKL  390

                                          400       410       420       430       440       450
Ce.thous                    IILSISYPTLSEVKGVIVHRLEAVSYNIGSQEWYTTVPRYVATNGYLISNFDESSCVFVSESAIC  455
C.penicillata20018          IILSISYPTLSEVKGVIVHRLEAVSYNIGSQEWYTTVPRYVATNGYLISNFDESSCVFVSESAIC  455
C.penicillatta21118         IILSISYPTLSEVKGVIVHRLEAVSYNIGSQEWYTTVPRYVATNGYLISNFDESSCVFVSESAIC  455
C.penicillatta6852          IILSISYPTLSEVKGVIVHRLEAVSYNIGSQEWYTTVPRYVATNGYLISNFDESSCVFVSESAIC  455
C.penicillatta6853          IILSISYPTLSEVKGVIVHRLEAVSYNIGSQEWYTTVPRYVATNGYLISNFDESSCVFVSESAIC  455
C.sp. Rio Grande do Norte   IILSISYPTLSEVKGVIVHRLEAVSYNIGSQEWYTTVPRYVATNGYLISNFDESSCVFVSESAIC  395
M.mulatta                   IILSISYPTLSEVKGVIVHRLEAVSYNIGSQEWYTTVPKYVATNGYLISNFDESSCVFVSESAIC  455
M.fascicularis              IILSISYPTLSEVKGVIVHRLEAVSYNIGSQEWYTTVPKYVATNGYLISNFDESSCVFVSESAIC  455
M.fascicularis              IILSISYPTLSEVKGVIVHRLEAVSYNIGSQEWYTTVPKYVATNGYLISNFDESSCVFVSESAIC  455
Ca.lupus Federal District   IILSISYPTLSEVKGVIVHRLEAVSYNIGSQEWYTTVPRYVATNGYLISNFDESSCVFVSESAIC  455

                                460       470       480       490       500       510       520
Ce.thous                    SQNSLYPMSPLLQQCIRGDTSSCARTLVSGTMGNKFILSKGNIVANCASILCKCYSTSTIINQSP  520
C.penicillata20018          SQNSLYPMSPLLQQCIRGDTSSCARTLVSGTMGNKFILSKGNIVANCASILCKCYSTSTIINQSP  520
C.penicillatta21118         SQNSLYPMSPLLQQCIRGDTSSCARTLVSGTMGNKFILSKGNIVANCASILCKCYSTSTIINQSP  520
C.penicillatta6852          SQNSLYPMSPLLQQCIRGDTSSCARTLVSGTMGNKFILSKGNIVANCASILCKCYSTSTIINQSP  520
C.penicillatta6853          SQNSLYPMSPLLQQCIRGDTSSCARTLVSGTMGNKFILSKGNIVANCASILCKCYSTSTIINQSP  520
C.sp. Rio Grande do Norte   SQNSLYPMSPLLQQCIRGDTSSCARTLVSGTMGNKFILSKGNIVANCASILCKCYSTSTIINQSP  460
M.mulatta                   SQNSLYPMSPILQQCIRGDTSSCARTLVSGTIGNKFILSKGNIVANCASILCKCYSTGTIINQSP  520
M.fascicularis              SQNSLYPMSPILQQCIRGDTSSCARTLVSGTIGNKFILSKGNIVANCASILCKCYSTGTIINQSP  520
M.fascicularis              SQNSLYPMSPILQQCIRGDTSSCARTLVSGTIGNKFILSKGNIVANCASILCKCYSTGTIINQSP  520
Ca.lupus Federal District   SQNSLYPMSPLLQQCIRGDTSSCARTLVSGTMGNKFILSKGNIVANCASILCKCYSTSTIINQSP  520

                                      530       540       550       560       570       580
Ce.thous                    DKLLTFIASDTCPLVEIDGITIQVGGRQYPDMVYESKVALGPAISLERLDVGTNLGNALKKLDDA  585
C.penicillata20018          DKLLTFIASDTCPLVEIDGITIQVGGRQYPDMVYESKVALGPAISLERLDVGTNLGNALKKLDDA  585
C.penicillatta21118         DKLLTFIASDTCPLVEIDGITIQVGGRQYPDMVYESKVALGPAISLERLDVGTNLGNALKKLDDA  585
C.penicillatta6852          DKLLTFIASDTCPLVEIDGITIQVGGRQYPDMVYESKVALGPAISLERLDVGTNLGNALKKLDDA  585
C.penicillatta6853          DKLLTFIASDTCPLVEIDGITIQVGGRQYPDMVYESKVALGPAISLERLDVGTNLGNALKKLDDA  585
C.sp. Rio Grande do Norte   DKLLTFIASDTCPLVEIDGVTIQVGGRQYPDMVYESKVALGPAISLERLDVGTNLGNALKKLDDA  525
M.mulatta                   DKLLTFIASDTCPLVEIDGVTIQVGGRQYPDMVYESKVALGPAISLERLDVGTNLGNALKKLDDA  585
M.fascicularis              DKLLTFIASDTCPLVEIDGVTIQVGGRQYPDMVYESKVALGPAISLERLDVGTNLGNALKKLDDA  585
M.fascicularis              DKLLTFIASDTCPLVEIDGVTIQVGGRQYPDMVYESKVALGPAISLERLDVGTNLGNALKKLDDA  585
Ca.lupus Federal District   DKLLTFIASDTCPLVEIDGVTIQVGGRQYPDMVYESKVALGPAISLERLDVGTNLGNALKKLDDA  585

                                590       600       610       620       630       640       650
Ce.thous                    KILIDSSNQILETVRRSSFNFGSLLSVPILICTALALLFLIYCCKRRYQQTLKQRTKVEPTFKPD  650
C.penicillata20018          KILIDSSNQILETVRRSSFNFGSLLSVPILICTALALLFLIYCCKRRYQQTLKQRTKVEPTFKPD  650
C.penicillatta21118         KILIDSSNQILETVRRSSFNFGSLLSVPILICTALALLFLIYCCKRRYQQTLKQRTKVEPTFKPD  650
C.penicillatta6852          KILIDSSNQILETVRRSSFNFGSLLSVPILICTALALLFLIYCCKRRYQQTLKQRTKVEPTFKPD  650
C.penicillatta6853          KILIDSSNQILETVRRSSFNFGSLLSVPILICTALALLFLIYCCKRRYQQTLKQRTKVEPTFKPD  650
C.sp. Rio Grande do Norte   KILIDSSNQILETVRRSSFNFGSLLSVPILICTALALLFLIYCCKRRYQQTLKQHTKVDPTFKPD  590
M.mulatta                   KVLIDSSNQILETVKRSSFNFGSMLSVPILICTALALLLLIYCCKRRYRQTFKHNTKVDPTFKPD  650
M.fascicularis              KVLIDSSNQILETVKRSSFNFGSMLSVPILICTALALLLLIYCCKRRYRQTFKHNTKVDPTFKPD  650
M.fascicularis              KVLIDSSNQILETVKRSSFNFGSMLSVPILICTALALLLLIYCCKRRYRQTFKHNTKVDPTFKPD  650
Ca.lupus Federal District   KILIDSSNQILETVRRSSFNFGSLLSIPILICTALALLFLIYCCKRRYQQTLKQHTKVEPTFKPD  650

                                      660
Ce.thous                    LTGTSKSYVRSL  662
C.penicillata20018          LTGTSKSYVRSL  662
C.penicillatta21118         LTGTSKSYVRSL  662
C.penicillatta6852          LTGTSKSYVRSL  662
C.penicillatta6853          LTGTSKSYVRSL  662
C.sp. Rio Grande do Norte   LTGTSKSYVRSL  602
M.mulatta                   LTGTSKSYVRSL  662
M.fascicularis              LTGTSKSYVRSL  662
M.fascicularis              LTGTSKSYVRSL  662
Ca.lupus Federal District   LTGTSKSYVRSL  662
```

X non-conserved

X ≥50% conserved

X all match
