# Supplementary material for: Emergence of a Novel Canine Distemper Virus Variant in Urbanized Free-Ranging Marmosets (Callithrix penicillata)
Source: Transbound Emerg Dis. 2025 Aug 15;2025:4818076. doi: 10.1155/tbed/4818076 (PMC12373474; doi:10.1155/tbed/4818076)
Supplement: Supporting Information 3 — S3. Multiple sequence alignment between P encoded from nonhuman primate-derived and canid-derived CDV isolates. From top to bottom, the Genbank Accession Numbers for each sequence are: XCJ77517.1, XCJ77509.1, XCJ77525.1, XCJ77533.1, XCJ77542.1, XJQ60209.1, ADN86309.1, BAM15596.1, BAM15588.2, and QSV52432.1. [file 4818076.f3.pdf]

```
                            10        20        30        40        50        60
Ce.thous                    MAEEQAYHVSKGLECLKALRENPPDLEEIQEVSSIRDQTRNPGQENGTAGMQEEEVSQDLDESHE  65
C.penicillata20018          MAEEQAYHVSKGLECLKALRENPPDLEEIQEVSSIRDQTRNPGQENGTAGMQEEEVSQDLDESHE  65
C.penicillatta21118         MAEEQAYHVSKGLECLKALRENPPDLEEIQEVSSIRDQTRNPGQENGTAGMQEEEVSQDLDESHE  65
C.penicillatta6852          MAEEQAYHVSKGLECLKALRENPPDLEEIQEVSSIRDQTRNPGQENGTAGMQEEEVSQDLDESHE  65
C.penicillatta6853          MAEEQAYHVSKGLECLKALRENPPDLEEIQEVSSIRDQTRNPGQENGTAGMQEEEVSQDLDESHE  65
C.sp. Rio Grande do Norte   MAEEQAYHVSKGLECLKSLRENPPDLEEIQEVSSIRDKTRNPDQQNGIASMQEEEVSQNLDESHE  65
M.mulatta                   MAEEQAYHVNKGLECLKALRENPPDIEEIQEVSSIRDQTRNPGQENGTASMQEEEVSQDLDESHE  65
M.fascicularis              MAEEQAYHVNKGLECLKALRENPPDIEEIQEVSSIRDQTRNPGQENGTASMQEEEVSQDLDESHE  65
M.fascicularis              MAEEQAYHVNKGLECLKALRENPPDIEEIQEVSSIRDQTRNPGQENGTASMQEEEVSQDLDESHE  65
Ca.lupus Federal District   MAEEQAYHVSKGLECLKALRENPPDLEEIQEVSSIRDQTCNPGQENGTASMQEEEVSQDLDESHE  65

                                 70        80        90        100       110       120       130
Ce.thous                    PAKGSNSVGHVLQNNPGSGKGNTALVEAEQPAKDVIQPGPGIRCYHVYDHSGEEVKGIEDADSLV  130
C.penicillata20018          PAKGSNYVGHVLQNNPGSGKGNTAPVEAEQPAKDVIQPGPGIRCYHVYDHSGEEVKGIEDADSLV  130
C.penicillatta21118         PAKGSNYVGHVLQNNPGSGKGNTAPVEAEQPAKDVIQPGPGIRCYHVYDHSGEEVKGIEDADSLV  130
C.penicillatta6852          PAKGSNYVGHVLQNNPGSGKGNTAPVEAEQPAKDVIQPGPGIRCYHVYDHSGEEVKGIEDADSLV  130
C.penicillatta6853          PAKGSNYVGHVLQNNPGSGKGNTAPVEAEQPAKDVIQPGPGIRCYHVYDHSGEEVKGIEDADSLV  130
C.sp. Rio Grande do Norte   PAKGSNYVGHVLQNNPGSGKGNTAPVEGEQPAKDVIQPGPGIRCYHVYDHSGEEVKGIEDADSLV  130
M.mulatta                   PAKGSNYLGHVLQNNPGCGESNTALVEAEQPAKDDIQPGPGIRCYHVYDHSGEEVKGIEDADSLV  130
M.fascicularis              PTKGSNYLGHVLQNNPGCGESNTALVEAEQPAKDDIQPGPGIRCYHVYDHSGEEVKGIEDADSLV  130
M.fascicularis              PTKGSNYLGHVLQNNPGCGESNTALVEAEQPAKDDIQPGPGIRCYHVYDHSGEEVKGIEDADSLV  130
Ca.lupus Federal District   PAKGSNYVGHVLQNNPGSGKGNTALVEAEQPAKDAIQPGPGIRCYHVYDHSGEEVKGIEDADSLV  130

                                    140       150       160       170       180       190
Ce.thous                    VPAGAVSNRGFERGEGSLDDSTEDSGEDYSEGNASSNWGYSFGLKPDRAADVSMLMEEELSALLK  195
C.penicillata20018          VPAGAVSNRGFERGEGSLDDSTEDSGEDYSEGNASSNWGYSFGLKPDRAADVSMLMEEELSALLK  195
C.penicillatta21118         VPAGAVSNRGFERGEGSLDDSTEDSGEDYSEGNASSNWGYSFGLKPDRAADVSMLMEEELSALLK  195
C.penicillatta6852          VPAGAVSNRGFERGEGSLDDSTEDSGEDYSEGNASSNWGYSFGLKPDRAADVSMLMEEELSALLK  195
C.penicillatta6853          VPAGAVSNRGFERGEGSLDDSTEDSGEDYSEGNASSNWGYSFGLKPDRAADVSMLMEEELSALLK  195
C.sp. Rio Grande do Norte   VPAGAVSNRGFERREGSLDDSTEDSGEDYSEGNASSNWGYSSGLKPDRAADVSMLMEEELSALLK  195
M.mulatta                   VPAGAVSNRGFERGEGSPDDSTEDSGEDYSEGNASSNWGYSFGLKPDRAADVSMLMEEELSALLK  195
M.fascicularis              VPAGAVSNRGFERGEGSPDDSTEDSGEDYSEGNASSNWGYSFGLKPDRAADVSMLMEEELSALLK  195
M.fascicularis              VPAGAVSNRGFERGEGSPDDSTEDSGEDYSEGNASSNWGYSFGLKPDRAADVSMLMEEELSALLK  195
Ca.lupus Federal District   VPAGAVSNRGFERGEGSLDDSTEDSGEDYSEGNASSNWGYSFGLKPDRAGDVSMLMEEELSALLK  195

                                200       210       220       230       240       250       260
Ce.thous                    TSRNVGIQKRDGKTLQFPHNPEGKIGDPECGSIKKGTGERSASHGMEIVAGSTNGATQSALKSTG  260
C.penicillata20018          TSRNVGIQKRDGKTLQFPHNPEGKIGDPECGSIKKGTGERSASHGMEIVAGSTNGATQSALKSTG  260
C.penicillatta21118         TSRNVGIQKRDGKTLQFPHNPEGKIGDPECGSIKKGTGERSASHGMEIVAGSTNGATQSALKSTG  260
C.penicillatta6852          TSRNVGIQKRDGKTLQFPHNPEGKIGDPECGSIKKGTGERSASHGMEIVAGSTNGATQSALKSTG  260
C.penicillatta6853          TSRNVGIQKRDGKTLQFPHNPEGKIGDPECGSIKKGTGERSASHGMEIVAGSTNGATQSALKSTG  260
C.sp. Rio Grande do Norte   TSRNVGIQKRDGKTLQFPHSPEGKTGDPECGSIKKGTGGRSASHGMEIVAGSTNGATQSALKSTG  260
M.mulatta                   TSRNVGIKKRDGKTLQFPHNPEGKTEDPECGSIKKGTGERSASHGMGIVAGSTNGATQSALKSTG  260
M.fascicularis              TSRNVGIKKRDGKTLQFPHNPEGKTEDPECGSIKKGTGERSASHGMGIVAGSTNGATQSALKSTG  260
M.fascicularis              TSRNVGIKKRDGKTLQFPHNPEGKTEDPECGSIKKGTGERSASHGMGIVAGSTNGATQSALKSTG  260
Ca.lupus Federal District   TSRNVGIQKRDGKTLQFPHNPEGKTGDPECGSIKKGTGERSASHGMEIVAGSTNGATQSALKSTG  260

                                     270       280       290       300       310       320
Ce.thous                    GSSGPSVSAENVRQPAVSAKMTQKCKPESGTQLPPKTSNEAETDSEYDDELFSEIQEIRSAITKL  325
C.penicillata20018          GSSGPSVSAENVRQPAVSAKMTQKCKPESGTQLPPKTSNEAETDSEYDDELFSEIQEIRSAITKL  325
C.penicillatta21118         GSSGPSVSAENVRQPAVSAKMTQKCKPESGTQLPPKTSNEAETDSEYDDELFSEIQEIRSAITKL  325
C.penicillatta6852          GSSGPSVSAENVRQPAVSAKMTQKCKPESGTQLPPKTSNEAETDSEYDDELFSEIQEIRSAITKL  325
C.penicillatta6853          GSSGPSVSAENVRQPAVSAKMTQKCKPESGTQLPPKTSNEAETDSEYDDELFSEIQEIRSAITKL  325
C.sp. Rio Grande do Norte   GSSGPSVSAENVRQPAVSAKMTQKCKPESGTQLPPKTPNEAESDSEYDDELFSEIQEIRSAITKL  325
M.mulatta                   GSSGPSVSAENVRQPAMNAKMTQKCKLESGMQLPPKTSNEAESDSEYDDELFSEIQEIRSAITKL  325
M.fascicularis              GSSGPSVSAENVRQPAMSAKMTQKCKPESGMQLPPKTSNEAESDSEYDDELFSEIQEIRSAITKL  325
M.fascicularis              GSSGPSVSAENVRQPAMSAKMTQKCKPESGMQLPPKTSNEAESDSEYDDELFSEIQEIRSAITKL  325
Ca.lupus Federal District   GSSGPSVSAENVRQPAMSAKMTQKCKPESGMQLPPKTSNEAETDSEYDDELFSEIQEIRSAITKL  325

                                 330       340       350       360       370       380       390
Ce.thous                    TEDNQAILSKLDTLLLLKGETDSIKKQISKQNIAISTIEGHLSSIMIAIPGFGKDTGDPTANVDI  390
C.penicillata20018          TEDNQAILSKLDTLLLLKGETDSIKKQISKQNIAISTIEGHLSSIMIAIPGFGKDTGDPTANVDI  390
C.penicillatta21118         TEDNQAILSKLDTLLLLKGETDSIKKQISKQNIAISTIEGHLSSIMIAIPGFGKDTGDPTANVDI  390
C.penicillatta6852          TEDNQAILSKLDTLLLLKGETDSIKKQISKQNIAISTIEGHLSSIMIAIPGFGKDTGDPTANVDI  390
C.penicillatta6853          TEDNQAILSKLDTLLLLKGETDSIKKQISKQNIAISTIEGHLSSIMIAIPGFGKDTGDPTANVDI  390
C.sp. Rio Grande do Norte   TEDNQAILSKLDTLLLLKGETDSIKKQISKQNIAISTIEGHLSSILIAIPGSGKDTGDPTANVDI  390
M.mulatta                   TEDNQAILSKLDTLLLLKGETDSIKKQISKQNIAISTIEGHLSSIMIAIPGFGKDTGDPTANVDI  390
M.fascicularis              TEDNQAILSKLDTLLLLKGETDSIKKQISKQNIAISTIEGHLSSIMIAIPGFGKDTGDPTANVDI  390
M.fascicularis              TEDNQAILSKLDTLLLLKGETDSIKKQISKQNIAISTIEGHLSSIMIAIPGFGKDTGDPTANVDI  390
Ca.lupus Federal District   TEDNQAILSKLDTLLLLKGETDSIKKQISKQNIAISTIEGHLSSIMIAIPGFGKDTGDPTANVDI  390

                                     400       410       420       430       440       450
Ce.thous                    NPELRPIIGRDSGRALAEVLKQPASSRGNRKDSGIALGSKGQLLRDLQLKPIDRESSSAIGYKPK  455
C.penicillata20018          NPELRPIIGRDSGRALAEVLKQPASSRGNRKDSGIGLGSKGQLLRDLQLKPIDRESSSAIGYKPK  455
C.penicillatta21118         NPELRPIIGRDSGRALAEVLKQPASSRGNRKDSGIGLGSKGQLLRDLQLKPIDRESSSAIGYKPK  455
C.penicillatta6852          NPELRPIIGRDSGRALAEVLKQPASSRGNRKDSGIGLGSKGQLLRDLQLKPIDRESSSAIGYKPK  455
C.penicillatta6853          NPELRPIIGRDSGRALAEVLKQPASSRGNRKDSGIGLGSKGQLLRDLQLKPIDRESSSAIGYKPK  455
C.sp. Rio Grande do Norte   NPELRPIIGRDSGRALAGVLKQPAASRGNKKDSGIALGSKGQLLRDLQLKPIDRESSSAIGYKPK  455
M.mulatta                   NPELRPIIGRDSGRALAEVLKQPASSRGNRKDGGIALGSKGQLLRDLQLKPIDKESSSAIGYKPK  455
M.fascicularis              NPELRPIIGRDSGRALAEVLKQPASSRGNRKDGGIALGSKGQLLRDLQLKPIDKESSSAIGYKPK  455
M.fascicularis              NPELRPIIGRDSGRALAEVLKQPASSRGNRKDGGIALGSKGQLLRDLQLKPIDKESSSAIGYKPK  455
Ca.lupus Federal District   NPELRPIIGRDSGRALAEVLKQPASSRGNRKDSGIALGSKGQLLRDLQLKPIDRESSSAIGYKPK  455

                                 460       470       480       490       500
Ce.thous                    DTAPSKAVLASLIRSSRVDQSHKHNMLALLKNIKGDDNLNEFYQMIKSITHV  507
C.penicillata20018          DTAPSKAVLASLIRSSRVDQSHKHNMLALLKNIKGDDNLNEFYQMIKSITHV  507
C.penicillatta21118         DTAPSKAVLASLIRSSRVDQSHKHNMLALLKNIKGDDNLNEFYQMIKSITHV  507
C.penicillatta6852          DTAPSKAVLASLIRSSRVDQSHKHNMLALLKNIKGDDNLNEFYQMIKSITHV  507
C.penicillatta6853          DTAPSKAVLASLIRSSRVDQSHKHNMLALLKNIKGDDNLNEFYQMIKSITHV  507
C.sp. Rio Grande do Norte   DTAPSKAVLASLIRSSRVDQSHKHNMLALLKNIKGDDNLTEFYQMIKSITHA  507
M.mulatta                   DTAPSKAVLASLIRSSRIDQSHKHNMLALLKNIKGDDNLNEFYQMIKSITHA  507
M.fascicularis              DTAPSKAVLASLIRSSRIDQSHKHNMLALLKNIKGDDNLNEFYQMIKSITHA  507
M.fascicularis              DTAPSKAVLASLIRSSRIDQSHKHNMLALLKNIKGDDNLNEFYQMIKSITHA  507
Ca.lupus Federal District   DTAPSKAVLASLIRSSRVDQSHKHNMLALLKNIKGDDNLNEFYQMIKSITHA  507
```

X non-conserved
X ≥ 50% conserved
X all match
